# Why crying does and sometimes does not seem to alleviate mood: a quasi-experimental study

**DOI:** 10.1007/s11031-015-9507-9

**Published:** 2015-08-23

**Authors:** Asmir Gračanin, Ad J. J. M. Vingerhoets, Igor Kardum, Marina Zupčić, Maja Šantek, Mia Šimić

**Affiliations:** TS Social and Behavioral Sciences, Department of Medical and Clinical Psychology, Tilburg University, PO Box 90153, 5000 LE Tilburg, The Netherlands; TS Humanities, Department of Communication and Information Sciences, Tilburg, The Netherlands; Department of Psychology, Faculty of Humanities and Social Sciences, University of Rijeka, Sveučilišna avenija 4, 51000 Rijeka, Croatia

**Keywords:** Emotional crying, Mood dynamics, Quasi-experiment

## Abstract

Whereas retrospective studies suggest that crying can be beneficial in terms of mood enhancement, results of quasi-experimental laboratory studies consistently demonstrate its negative effects on mood. The present study was specifically designed to evaluate a parsimonious explanation for this paradox by assessing mood after crying in a laboratory, both immediately and at follow up. Mood ratings of 28 objectively established criers and 32 non-criers were compared before and immediately after the exposure to an emotional movie, as well as 20 and 90 min later. As expected, immediately after the film, negative mood significantly increased in criers, while it did not change in non-criers. This mood deterioration was followed by a recovery that resulted in return to the baseline mood levels at the third measurement. Criers subsequently reported mood enhancements at the final measurement compared to the pre-film measurement. Crying frequency did not predict mood changes above those predicted by the presence of crying. The observed relation between crying and more long-term mood recovery reconciles seemingly contrasting earlier results and provides a simple and obvious explanation. After the initial deterioration of mood following crying that was observed in laboratory studies, it apparently takes some time for the mood, not just to recover, but also to become even less negative than before the emotional event, which corresponds to the results of retrospective studies.

## Introduction

Humans are the only species having the capacity to shed emotional tears, a behavior that is very common and present in all cultures (Trimble 2012; Vingerhoets 2013; Vingerhoets and Bylsma 2015). Nevertheless, it has received little attention of researchers and the understanding of its functions is still very limited. Theories on the functions of crying can be classified into two global categories (Vingerhoets 2013; Vingerhoets et al. 2009). The first one emphasizes the putative *intra*-individual benefits of crying, the catharsis effect. According to this view, crying facilitates recovery and homeostatic processes. The alternative view emphasizes the *inter*-individual functions of crying, which are represented in the communication of one’s helplessness and need for support, and whose effects are evident in elicitation of comfort and succor (Hasson 2009). These two positions are not mutually exclusive but rather supplemental, because receiving comfort and emotional support may also contribute to an increased well-being after crying. Seen from a stress theoretical perspective, crying may be considered as a kind of coping behavior, serving several functions, ranging from facilitation of emotional recovery to an ultimate effort to persuade others to do something about the situation (Vingerhoets 2013).

The empirical support for the notion that crying indeed facilitates recovery and brings relief is mixed (Cornelius 1997; Gračanin et al. 2014; Rottenberg et al. 2008a). Rottenberg et al. (2008b) after reviewing the relevant literature came to conclude that the question “Does crying bring relief?” is not adequate, but rather that the better alternative is “For whom, and under which specific conditions does crying bring relief?” More precisely, person characteristics, the nature of the specific crying antecedent, and the reactions of others all seem to determine how people feel after having cried. These authors further emphasize that the research findings also depend on the nature and design of the study. Surveys addressing general opinions about the effects of crying on mood and retrospective self-report studies consistently yield support for the claim that crying is generally followed by emotional relief, and that it results in decreases in negative and increases in positive mood (e.g., Bindra 1972; Rottenberg et al. 2008b; but see Labott and Martin 1988; for reviews see Cornelius 1997; Vingerhoets 2013). In contrast, laboratory studies, in which crying is induced by exposing the participants to emotional films, show consistent decreases in mood immediately after the film (e.g., Gross et al. 1994; Kraemer and Hastrup 1988; Martin and Labott 1991; for an overview see Cornelius 1997).

When trying to explain these seemingly discrepant findings, some critical methodological issues need to be taken into account. In retrospective studies, participants are generally free to choose which crying episodes they describe. It has been suggested that such a design leaves the possibility that participants preferred to report crying experiences that fit the popular notion that one feels better after having cried (Cornelius 1997). An additional problem in these retrospective studies is that it is not possible to precisely define the time interval between the crying episode and the reported feeling. Participants are usually asked to report how they feel after having cried, without specification how they felt immediately, 10 min or 1 h after crying. One may, of course, wonder whether that would make any sense, that is, whether it is possible for participants to remember exactly the time course of their mood changes. However, it nevertheless could be that, after having cried, people typically first experience a dip in their mood, which is subsequently logically followed by a mood improvement. We currently simply lack the necessary information about the precise time course of mood changes after crying that would help us to understand the popular postulate of emotional recovery that follows tears.

In most of the laboratory studies, mood was measured immediately after the film that was used to induce crying, which certainly makes the time range for mood improvements strictly limited in comparison to that in retrospective studies. In only three studies (Kraemer and Hastrup 1988; Labott and Martin 1988; Martin and Labott 1991) attention has been paid to possible delayed effects of the crying episode on subsequent mood. Results of these studies showed no differences in experienced stress (Labott and Martin 1988) or depressive mood (Kraemer and Hastrup 1988) at later points in time following a crying episode, or they showed deteriorating effects of crying on mood (Martin and Labott 1991). However, these studies were characterized by a short time interval (15 min) between the crying episode and the final mood measurement (Martin and Labott 1991), a significant drop out of participants at the follow up measurement, lack of control over the timing of the last measurement (Labott and Martin 1988), and an artificial condition in which participants were instructed either to cry or to suppress crying (Kraemer and Hastrup 1988), without an objective check of their compliance.

The present study has been especially designed to examine the immediate and delayed effects of crying on mood in a laboratory setting. To increase generalizability and validity, we decided to use stimuli that provoke tears through a wide spectrum of negative and positive emotions. In accordance with the results obtained in previous laboratory studies (Cornelius 1997), we predicted that increases in negative mood (here termed as negative affect, NA) immediately after the potential crying–eliciting films would be more pronounced in participants who cried than in participants who did not cry. Furthermore, based on the results of previous retrospective studies, we anticipated that at the delayed mood measurements, participants who cried would experience greater mood improvement compared to participants who did not cry, both in terms of recovery after initial mood deterioration and of overall mood improvement (i.e., compared to the pre-film baseline values). We also anticipated the presence of a dose–response relationship between frequency of crying episodes and the changes in NA during the same time sequences.

## Method

### Participants

The initial sample consisted of 46 female and 26 male students (age range 19–33; *M* = 23.80; *SD* = 3.19) who received course credits for participation. All participants provided written informed consent. Six participants were excluded from the analyses because of missing behavioural data due to equipment failure or because of incomplete questionnaire data.

### Tools

#### Film stimuli

As cry eliciting stimuli we used two films that, in a pilot study among 102 participants, provoked a wide range of different emotions resulting in crying. Both films, edited in such a way that they retained all the most dramatic scenes that were important for the elicitation of emotions and crying, included four pre-defined scenes each of which produced crying (self-reports) in at least 10 % of the participants of the pilot study. The films included at least one scene that induced crying in more than 30 % of participants and also contained a maximum of five potential additional tear-eliciting scenes with a low frequency of elicited crying. These latter scenes were retained because (a) of their importance in predisposing the participants for the elicitation of crying in subsequent scenes, and (b) they should have increased inter-individual variability in crying frequency that we were interested in. During the edited version of *La vita è bella* (Life is beautiful; Benigni 1997; 46′23″ long) 40 % participants reported crying (18, 10, 16 and 33 % of participants during the four pre-planned scenes, respectively) and during the edited version of *Hachi: A dog’s Tale* (Hallström 2009; 45′32″ long) crying was reported in 69 % of the participants (35, 38, 65, and 58 % of participants during the four pre-planned scenes, respectively) in the pilot study. The first three pre-planned cry eliciting scenes appeared 25, 15, and 5 min and 15, 12, and 5 min before the end of the films, respectively. The last cry eliciting scenes in both films were presented at the very end of each film. Therefore, the most of the participants that cried, did so during the very last part of each film.

#### Mood scale

NA was assessed using the shortened version of the *Emotional States Scale* (Kardum and Bezinović 1992), containing 18 items on a five-point Likert scale ranging from 1 (I do not feel this way at all) to 5 (I feel this way completely). The items were: nervous, bad tempered, anxious, weepy, tense, guilty, helpless, sad, angry, miserable, rejected, fearful, cheerful, generous, relaxed, calm, active and merry (last six items reversely keyed). The scale showed adequate internal consistency, with the Cronbach’s alphas over the four measurements ranging from .77 to .84.

#### Detection of crying episodes

In order to measure crying behavior, video recordings of the participants’ eyes were made and analyzed. Two unobtrusive side light sources were directed at the participant’s face in order to make the changes in moistening of the eyes better visible. Two coders independently rated the degree of participants’ eye moistening during each of the four targeted scenes as well as during any of the five additional scenes for which any of the participants in the pilot study had indicated that it had evoked tears. The task of the coders was to indicate each time when the participants’ eyes started filling with tears. The two-way random absolute agreement between the two coders was moderate [ICC(_2,2_) = .692; 95 % CI .50–.81].

### Procedure

Participants were individually seated in a sound attenuated room approximately 60 cm from a 19 in. monitor, speakers and video-camera. After having signed the informed consent form, they completed the first mood rating (T1), which was immediately followed by the random exposure to one of the two films. After having watched the film, participants first filled in a short questionnaire about their emotional responses to films that is not in the scope of this study. This was followed by the second mood measurement (T2) after which they were accompanied by the experimenter to another room. After they had filled in additional questionnaires also not in the scope of this study, 20 min following T2 participants completed the mood questionnaire for the third time (T3). After being shortly debriefed, participants were given additional instructions and a closed envelope containing the mood scale which they were requested to complete and return immediately after having received an SMS message via mobile phone. Each participant left the laboratory 120 min after the beginning of the experiment. The fourth mood measurement (T4) took place 90 min after the second one and 60 min after the participants left the laboratory. Participants were not given any instructions how to behave during the 60 min after leaving the laboratory and before T4. The answers to the T4 measure were sent to the experimenter via text messages in which participants returned 18 numbers representing their responses on the mood scale items.

### Data analysis

To explore whether the participants who cried and who did not cry differed in changes in NA throughout the four mood measurements, groups were created according to the following criteria. A participant was assigned to the *crying* group if it was objectively, by the two coders, established that (s)he cried during at least one scene of the film, while the *non*-*crying* group consisted of participants who did not cry throughout the film presentation. For each scene to be coded as a crying scene both coders thus had to observe the appearance of tears. In order to keep the time interval between the crying and the mood assessment constant, we excluded the six participants for whom the last crying episode appeared earlier than the last scene in the film from all analyses. In addition to a better control of the time between crying episodes and mood measurements, such a selection was important because the individuals that cried earlier but not at the end of the film faced different conditions after their crying episodes in comparison to other participants, due to the expected emotional impact of the final parts of the films.

First, independent samples *t* test, Chi square tests, and Pearson correlation coefficients were calculated to test the relations between all the relevant variables, in order to test for the existence of possible confounding variables that have to be controlled for. Next, in order to examine whether the groups of participants who cried and who did not cry differed in changes in NA between the four measurements, a 4 × 2 mixed ANOVA was performed with time period as a within-subjects factor (measurements at T1, T2, T3 and T4), group (non-crying and crying) as between-subjects factor, and age and gender as covariates. Changes within the groups and between specific measurements were analyzed by comparison of NA between points in time for which the hypotheses were made: between (1) T1–T2 (expected mood deterioration), (2) T1 and T4 (expected overall mood enhancement), and (3) T2–T4 (expected recovery), by using two separate within-subjects ANOVAs (for each group), and Bonferroni post hoc tests. To validate the possibility that low mood in criers stimulated (successful) mood enhancing behaviors, we also performed an additional ANOVA by including NA change from T1 to T2 as a covariate.

In order to prepare the data for regression analyses, the *crying* measure was aggregated across all the scenes for which the presence of crying was observed, resulting in the variable *frequency of crying episodes* (FCE). To test if FCE predicted changes in NA between relevant points in time, three multiple regression analyses were conducted. In each analysis, age, gender, and NA at T1 were entered in the first block, while FCE was entered as predictor in the second block. Change scores for relevant time periods were entered as criterion variables. Here we report only the effects of the FCE (i.e., the second block). In order to avoid inflation of the effects of the variable *crying* (i.e., whether a participant cried or not), only the crying participants were included in these regression analyses.

## Results

During the film *La vita è bella* 45 % of those who watched that film have cried (6, 15, 12 and 41 % of participants during the four pre-planned scenes, respectively), and during the film *Hachi: A dog’s Tale* crying was observed in 57 % of the participants (0, 11, 43, and 43 % of participants during the four pre-planned scenes, respectively). The number of participants included in the analyses was 32 in the non-crying group (16 females and 16 males) and 28 in the crying group (23 females and 5 males). Descriptive data, correlations, and the differences between the crying and non-crying participants for all continuous variables are presented in Table 1. Significantly more females cried (*χ*^2^ = 6.78; *p* = .009) and, within the group of criers, females cried more often (*t* = 3.15; *p* = .006). Also, NA and NA changes from T1 to T2, T2 to T3, and T2 to T4 in females were higher (*t* = 2.74, *p* = .008; *t* = 2.74, *p* = .008; *t* = 2.21, *p* = .03; *t* = 2.48, *p* = .016, respectively). The type of film presented was not related to any of the variables. Given these results, gender and age were controlled for in all subsequent analyses.

**Table 1:** Bivariate correlations, means, standard deviations and differences between the crying and non-crying participants for all continuous variables

	1	2	3	4	5	6	7	8	9	10	11	Means (SDs)				
												All	Non-crying	Crying	*t*	*d*
1. T1	–											1.93 (.37)	1.99 (.38)	1.85 (.36)	1.47	.38
2. T2	.24	–										2.25 (.48)	2.13 (.43)	2.38 (.50)	2.06*	.54
3. T3	.51*	.56*	–									1.80 (.31)	1.79 (.27)	1.74 (.35)	.60	.16
4. T4	.45*	.29*	.47*	–								1.76 (.38)	1.81 (.44)	1.62 (.29)	1.94	.51
5. T2–T1	−.49*	.73*	.14	−.06	–							.72 (.53)	.14 (.49)	.53 (.50)	3.02*	.79
6. T3–T1	−.63*	.24*	.35*	−.07	.66*	–						−.16 (.34)	−.21 (.34)	−.12 (.35)	1.05	.26
7. T4–T1	−.50*	.05	−.03	.54*	.40*	.52*	–					−.21 (.40)	−.19 (.44)	−.23 (.35)	.41	.10
8. T3–T2	.11	−.76*	.11	.02	−.76*	−.01	−.08	–				−.49 (.40)	−.35 (.33)	−.64 (.42)	3.06*	.77
9. T4–T2	.11	−.71*	−.16	.48*	−.72*	−.27*	.35*	.72*	–			−.53 (.52)	−.33 (.48)	−.76 (.48)	3.50*	.90
10. T4–T3	.04	−.17	−.36*	.66*	−.19	−.37*	.59*	−.07	.64*	–		−.04 (.36)	.02 (.42)	−.12 (.29)	1.46	.39
11. FCE	−.15	−.29*	.00	−.21	.37*	.16	−.06	−.35*	−.42*	−.22	–	1.10 (1.47)	.00 (.00)	2.36 (1.28)	9.72*	2.61
12. Age	.04	−.30*	−.24	−.09	−.30*	−.26*	−.13	.17	.20	.10	.02	23.95 (3.14)	23.78 (3.32)	24.14 (2.82)	.44	.12

Standard deviations are in parentheses

*T1* pre-film negative affect; *T2* post-film negative affect; *T3* negative affect follow up at 20 min; *T4* negative affect follow up at 90 min; *FCE* frequency of crying episode

* Critical alpha level is set to *p* < .05

**Fig. 1:**
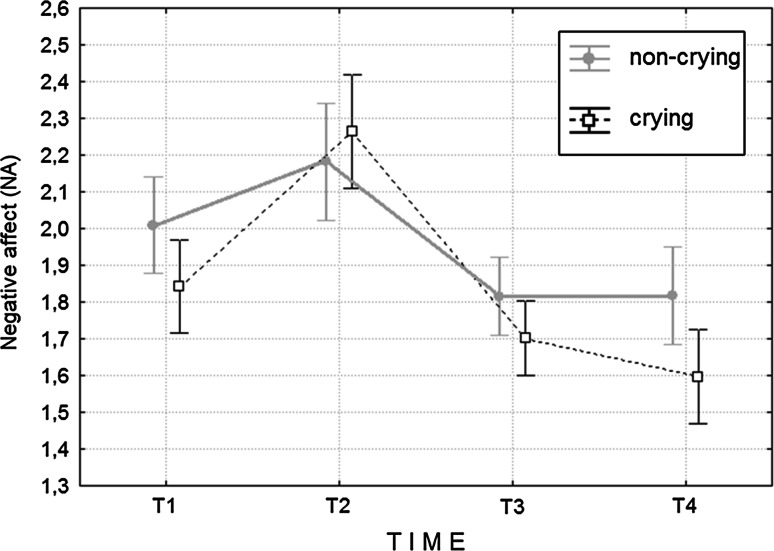
Means and SEs of NA in crying and non-crying groups at T1, T2, T3 and T4. *T1*, pre-film; *T2*, post-film; *T3*, follow up at 20 min; *T4*, follow up at 90 min

A significant main effect of time [F(3, 53) = 5.64; *p* = .002; partial η^2^ = .09] and a significant time × group interaction [F(2, 36) = 5.11; *p* = .003; partial η^2^ = .08] were found—see Fig. 1—confirming our hypothesis about the different course of mood changes in the crying and non-crying group. No main effect of the group [F(1, 55) = .83; *p* = .365; partial η^2^ = .02] was observed.

To better understand the observed interaction two separate ANOVAs were performed in order to test whether there was a significant mood change in each of the two groups (*p* set to .025). There was no significant overall change in NA in the group of non-criers throughout the four measurements [F(3, 27) = 1.20; *p* = .311; partial η^2^ = .04]. However, in the group of criers, a significant effect of time was observed [F(3, 23) = 3.95; *p* = .021; partial η^2^ = .34]. Post hoc comparisons (Bonferroni correction) revealed that NA increased from T1 to T2 (*p* < .001), and decreased from T2 to T3 and from T2 to T4 (*p* < .001), thus supporting our hypotheses about initial deterioration and subsequent recovery of mood in criers. Most importantly, however, the decrease in NA from T1 to T4 in this group was also significant (*p* = .013), thus supporting our hypothesis about the overall mood improvements following crying. Finally, the predicted decreases in NA remained significant (recovery: *p* < .001; overall mood improvement: *p* = .007) when NA change from T1 to T2 was included as a covariate in an additional ANOVA.

When the ANOVA was repeated by including the six participants who reported the last crying episode earlier than the last scene in the film, the overall change in mood became marginally significant in the crying group [F(3, 29) = 1.76; *p* = .093; partial η^2^ = .08], but all the hypothesized changes (T1–T2, T2–T4, and T1–T4) remained significant (*p* < .001, *p* < .001, and *p* = .003, respectively). Similar as in the previous analysis, no overall change in mood was observed in the non-crying group.

Regarding the possible dose–response relations between crying frequency and subsequent mood changes, FCE failed to explain the remaining variance of changes in NA from T1 to T2 (R^2^ = .38; ∆R^2^ = .01; β = .10; *p* = .572). Contrary to our hypothesis, NA did not increase from T1 to T2 more in those participants who reported more crying episodes. Also contrary to our hypotheses, changes in NA from T2 to T4 and from T1 to T4 were not related to FCE (R^2^ = .50; ∆R^2^ = .03; β = −.19; *p* = .333, R^2^ = .69; ∆R^2^ = .01; β = −.11; *p* = .489, respectively). In general, whether a participant cried or not seemed to be more important for the subsequent mood changes than the specific frequency of crying.

## Discussion

To our knowledge, this is the first study that more systematically and thoroughly investigated the effects of the experimental induction of crying on subsequent mood across several time points. The most important finding was that the participants who cried reported an increase in NA immediately after their crying as well as subsequent decreases in NA at follow up (i.e., 20 and 90 min post film) in comparison to the measurement immediately after the film and also when compared to the pre-film baseline measurement (only in the case of 90 min post film). In contrast, non-criers did not experience any mood changes after the film presentation. Thus, the present results seem to reconcile the findings of previous quasi-experimental studies of crying (i.e., that crying predominantly results in an increase in NA immediately after the films) and of retrospective studies (i.e., that crying can facilitate mood improvement). This is the first study that has demonstrated a clear relation between experimentally induced crying and subsequent, more long-term mood improvement. Regarding the hypothesized dose–response relationship, frequency of crying did not have any predictive power when only the crying participants were taken into account. Irrespective of how often the participants cried, the immediate decreases as well as subsequent increases in their mood were rather similar.

Perhaps it is in particular the strong mood improvement experienced by those participants who cried that fuels the popular notion that crying brings relief. It is obvious that decreases in NA from T2 to T4 and from T3 to T4 in participants who cried are mainly the consequences of previous (i.e., T1–T2) NA increases, that is, the return to baseline levels. This finding may explain why people report mood improvement after crying: those who cried indeed experience greater mood changes, be it after an initial deterioration. Such strong mood recovery, as well as the observed return of NA even to below baseline levels thus seems to support the hypothesis about the cathartic effects of crying. These findings are in accordance with both of our hypotheses about the negative effects of crying over the short run and about mood increases that follow crying over the longer run. On a more general level, these findings can be compared to the short-term negative and long-term positive effects of the expression of emotion on mood and well-being (Pennebaker 1997; Smyth 1998). However, the question remains whether these long-term mood-enhancing effects of crying and of other types of emotion expression are mediated by the same cognitive, physiological, or behavioral mechanisms.

One could argue that the observed general decline in NA reported by criers at the final measurement is the consequence of increased tension and nervousness of the study participants at the beginning of the experiment, due to the fact that they are in a new and uncertain situation, rather than being the result of crying. However, if this mood improvement is considered as a kind of return to the (pre-experiment) baseline level, it remains unclear why non-criers did not end up with improved mood at the final relative to the first measurement. Also note that both groups did not differ in NA at T1.

If the observed decline in NA below the baseline level reflects the real effects of crying, which physiological mechanisms could account for such finding? The most likely candidates are increases in parasympathetic activation and increases in levels of oxytocin, which may accompany specific cognitive and behavioral mechanisms (see Gračanin et al. 2014). For example, there is mounting evidence that crying is accompanied with and possibly followed by increases in parasympathetic activation (e.g., Hendriks et al. 2007; Rottenberg et al. 2003), which is also related to states of relaxation and absence of intensive negative emotion (Porges 2003). In addition, increases in oxytocin are thought to accompany crying, resulting not only from comforting behaviors that are elicited by crying, but also from behavioural and cognitive mechanisms that are not necessarily based on the reception of social support (Vingerhoets 2013; Gračanin et al. 2014). Since there is some evidence that low oxytocin is related to sadness (Turner et al. 1999), one could speculate that increases in the level of this hormone following crying could lead to associated decreases in NA. On the other hand, a much simpler explanation is that the decreased mood stimulates the participants to apply all kinds of cognitive and behavioral mood management strategies, which would result in the desired mood improvement, without any necessary influence of the here suggested, direct physiological and cognitive mechanisms, induced by the act of crying. However, after partializing out the effect of change in NA from T1 to T2, the observed decrease in NA remained significant. Thus, the amount of initial NA increase, that could have motivated participants to engage in mood enhancing behaviors, was not related to general mood improvement. The stability of this effect after excluding the effects of initial mood deterioration also contrasts the possibility that criers ended up with increased mood because they were generally more emotionally reactive, since the initial increase in NA did not seem to affect the mood improvement at the delayed measurement as compared to the measurement before crying.

Two aspects of this study were innovative. First, mood was assessed at three fixed times after having been exposed to an emotional film. Although the time of the initial crying episodes varied across participants, this variation was relatively small, and, more importantly, the time of the last crying episode was fixed. Second, objectively established crying was operationalized both in terms of mere appearance as well as in terms of frequency. The relatively high number of participants and the optimal ratio of criers to non-criers represent strong features of the study as well. On the other hand, we have to keep in mind that the present study, being similar to previous laboratory studies, also necessarily had a quasi-experimental design, meaning that participants were assigned to the two groups on the basis of their responses to the films. It thus cannot be excluded that individuals who did and did not cry, differed in certain personality traits (Stougie et al. 2004) or other, more temporarily characteristics related to changes in crying and/or mood after the exposure to emotional stimuli (cf. Vingerhoets 2013). For example, previous research has shown that more neurotic individuals cry more often (De Fruyt 1997) and more easily (Peter et al. 2001), while, for example, introverted and depressive individuals report more often the absence of mood benefits following crying (De Fruyt 1997; Rottenberg et al. 2008b). Kraemer and Hastrup (1988) tried to solve this issue of confounding individual differences by instructing participants to cry or to suppress their tears, but, as noted above, the adequacy of that solution is arguable because such a manipulation makes the situation rather artificial. Bylsma et al. (2011) applied a diary methodology in order to separate inter-individual from intra-individual differences in mood that follows crying. They showed that crying is related to increased negative mood even the day after the crying episode. Although this method has a high ecological validity, it still does not exclude the non-systematic effects of different conditions that inflict both crying and negative mood during the periods of several days. It is possible that both crying and mood dynamics observed in our as well as the latter study are influenced by some temperamental characteristics, such as emotional reactivity (Larsen and Diener 1987), despite the above presented preliminary findings which seem to challenge such an explanation. Relatedly, distinct mood dynamics of criers and non-criers in our study could be interpreted in the context of previous research showing a positive relation between emotion suppression and vulnerability to mood disturbances (Ehring et al. 2010).

A final critical and still unanswered question is whether the observed decreased NA really reflects a different mood state, induced by the crying, or that it reflects a kind of response shift, a phenomenon, which is well-known in the literature on quality of life in cancer patients. Repeatedly it has been found that cancer patients report a better quality of life than before their disease, when comparing their absolute scores on quality-of-life measures. However, when being asked to compare directly their current quality of life with before their disease (as is also done in retrospective studies on mood changes after crying), they indicate unanimously that their current state is worse than before their disease (Schwartz and Sprangers 1999). This response shift might be the result of changes in internal standards, conceptualization of the concepts, and/or one’s values. This methodological issue certainly needs adequate consideration in future studies, for example by including a measure asking participants to compare directly their current mood state to the pre-film measurement. For future studies, we also suggest to pay an additional attention to factors that influence the memory of emotional events and mood, to stable individual differences related to affect intensity and variability, as well as to mood regulation processes.

In conclusion, the present findings suggest a simple, obvious, and parsimonious explanation for the paradoxical findings of different studies investigating the effects of crying on mood. After the initial deterioration of mood following crying that is usually observed in laboratory studies, it takes some time for the mood, not just to recover, but also to increase above the levels that it had before the emotional event, a pattern of findings which corresponds to the results of retrospective studies.
